# Pituitary hemochromatosis in the clinical setting of secondary amenorrhea in a patient with Diamond-Blackfan anemia

**DOI:** 10.1016/j.radcr.2024.10.062

**Published:** 2024-11-08

**Authors:** Jason Chandrapal, David Fetzer, Vishal Kukkar, Fabricio Feltrin

**Affiliations:** a University of Texas Southwestern, 5323 Harry Hines Blvd, Dallas, TX 75390, USA; b Cleveland Clinic Foundation, 9500 Euclid Ave, Cleveland, OH 44195, USA

**Keywords:** Hemochromatosis, Secondary amenorrhea, Hypogonadism, Pituitary dysfunction, Anemia

## Abstract

Secondary amenorrhea is the absence of menses for more than 3 months in women who previously had regular menstrual cycles or 6 months for those with irregular cycles. Workup of secondary amenorrhea includes laboratory analysis to assess pituitary function, specifically luteinizing hormone (LH) and follicle stimulating hormone (FSH). If low, structural evaluation of the pituitary gland with MRI is recommended. We report a case of a 31-year-old female with history of transfusion-dependent Diamond-Blackfan anemia and type 2 diabetes that reported amenorrhea for 1 year following intrauterine device (IUD) removal. Due to low LH and FSH, the patient underwent an MRI of the pituitary gland. Imaging demonstrated complete absence of MRI signal within the pituitary parenchyma, which confirmed pituitary dysfunction from secondary hemochromatosis, presumably due to iron overload from multiple transfusions. As a result of her imaging and laboratory assessment, she was placed on an iron chelator and oral contraception.

## Introduction

Amenorrhea is the absence of menses and is caused by dysfunction of the hypothalamic-gonadal axis. Primary amenorrhea is defined as absence of menarche by age 15 or later and secondary amenorrhea is the absence of menses for more than 3 months in women who previously had regular menstrual cycles or 6 months for those with irregular cycles. While ovarian dysfunction is the most common cause of secondary amenorrhea, the hypothalamic-gonadal axis of the central nervous system (CNS) accounts for a significant proportion. In a case series of 262 adult patients, secondary amenorrhea was caused by ovarian, hypothalamic, and pituitary dysfunction in 40%, 35%, and 17% of patients, respectively [1].

The CNS influence starts with the hypothalamus, which releases gonadotropin-releasing hormone (GnRH) causing the downstream anterior pituitary gland to release follicle stimulating hormone (FSH) and luteinizing hormone (LH) into the system blood stream. These hormones transport the CNS signal directly to the ovaries which release progesterone and estrogen that stimulate menstrual cycle. Any alteration in this pathway can result in secondary amenorrhea and include nutritional deficiencies, medications that affect hormones, suprasellar masses, or primary pituitary abnormalities.

## Case report

A 31-year-old woman with a past medical history of transfusion-dependent Diamond-Blackfan anemia and type 2 diabetes mellitus, was seen in endocrine clinic for secondary amenorrhea after removal of her intrauterine device (IUD) one year prior. Since the removal of her IUD, she had not menstruated. The patient had irregular menses prior to placement and had the IUD for a duration of 3 years. She began menarche at 12 years old and her periods were also irregular at that time. An endometrial biopsy recently performed demonstrated an atrophic endometrium. On assessment the patient had normal vital signs and an unremarkable physical exam. Her laboratory assessment suggested a constellation of findings such as pancytopenia, elevated liver function enzymes, iron overload, and low FSH/LH and estradiol (Table 1).

**Table 1:** A 31-year-old female patient laboratory values.

Laboratory test	Patient's values	Normal values (*gender and age matched)
*CBC with differential:*		
WBC	1.67	4.5-11
Hemoglobin	7.5 g/dL	12-15.5 g/dL*
Hematocrit	23.4%	36%-48%*
Platelets	77	150-300
Mean corpuscular volume (MCV)	91 fl	80-100 fl
*Iron studies:*		
Iron	272 mcg/dL	50-170 mcg/dL*
Ferritin	15,170 ng/mL	24-307 ng/mL*
Total iron binding capacity (TIBC)	unable to calculate	240-450 mcg/dL
Unsaturated iron binding compacity (UBC)	<17 mcg/dL	111-343 mcg/dL
*Endocrine studies:*		
Thyroid stimulating hormone (TSH)	6.7 mIU/mL	0.4-4 mlU/L
Adrenocorticotropic hormone (ACTH)	21.6 pg/mL	10-60 pg/mL
Dehydroepiandrosterone-sulfate (DHEA-sulfate)	65 mcg/dL	45-270 mcg/dL*
Luteinizing hormone	18 IU/mL	5-25 IU/mL*
Follicle-stimulating hormone (FSH)	2.5 mIU/mL	4.7-21.5 mIU/mL*
Estradiol	<10 pg/mL	30-400 pg/mL*
Testosterone	31 ng/dL	15-70 ng/dL*

**Fig. 1:**
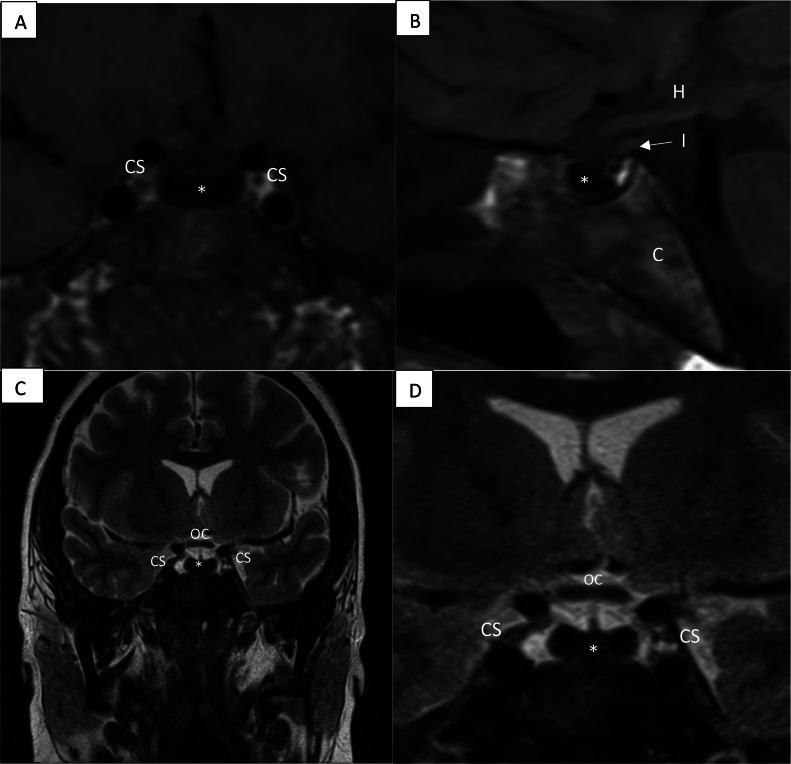
MRI of the suprasellar/pituitary region of the brain. T1 coronal (A) and sagittal (B) and T2 coronal images (C and D) with diffuse T1 and T2 signal loss within the pituitary gland (*). T1 signal of the normal pituitary gland should be isointense within the anterior segment and hyperintense in the posterior segment. For T2 sequences, the normal anterior and posterior pituitary should be isointense. The complete T1 and T2 signal loss in the pituitary is due to iron deposition from secondary hemochromatosis. The loss of signal intensity is lower than the adjacent bone signal. * = pituitary gland, CS = cavernous sinus, H = hypothalamus, I = infundibulum, OC = optic chiasm, C = clivus.

**Fig. 2:**
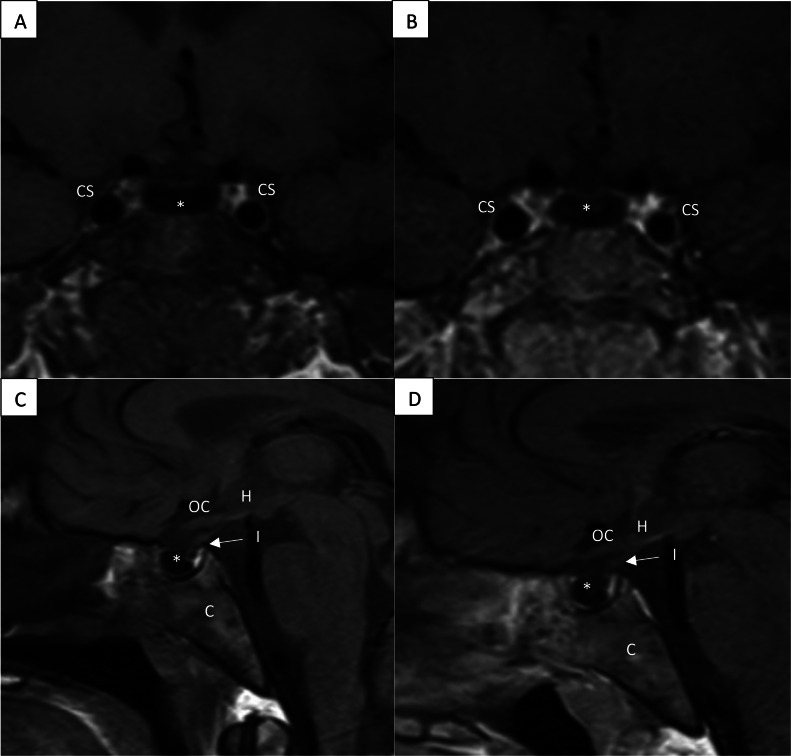
T1 pre- and post-contrast in coronal (A and B) and sagittal (C and D) images. Postcontrast T1 sequences demonstrate a lack of enhancement within the pituitary gland. Normally the pituitary gland should demonstrate T1 homogenous enhancement which is not seen due to excessive iron deposition from secondary hemochromatosis. * = pituitary gland, CS = cavernous sinus, H = hypothalamus, I = infundibulum, OC = optic chiasm, C = clivus.

**Fig. 3:**
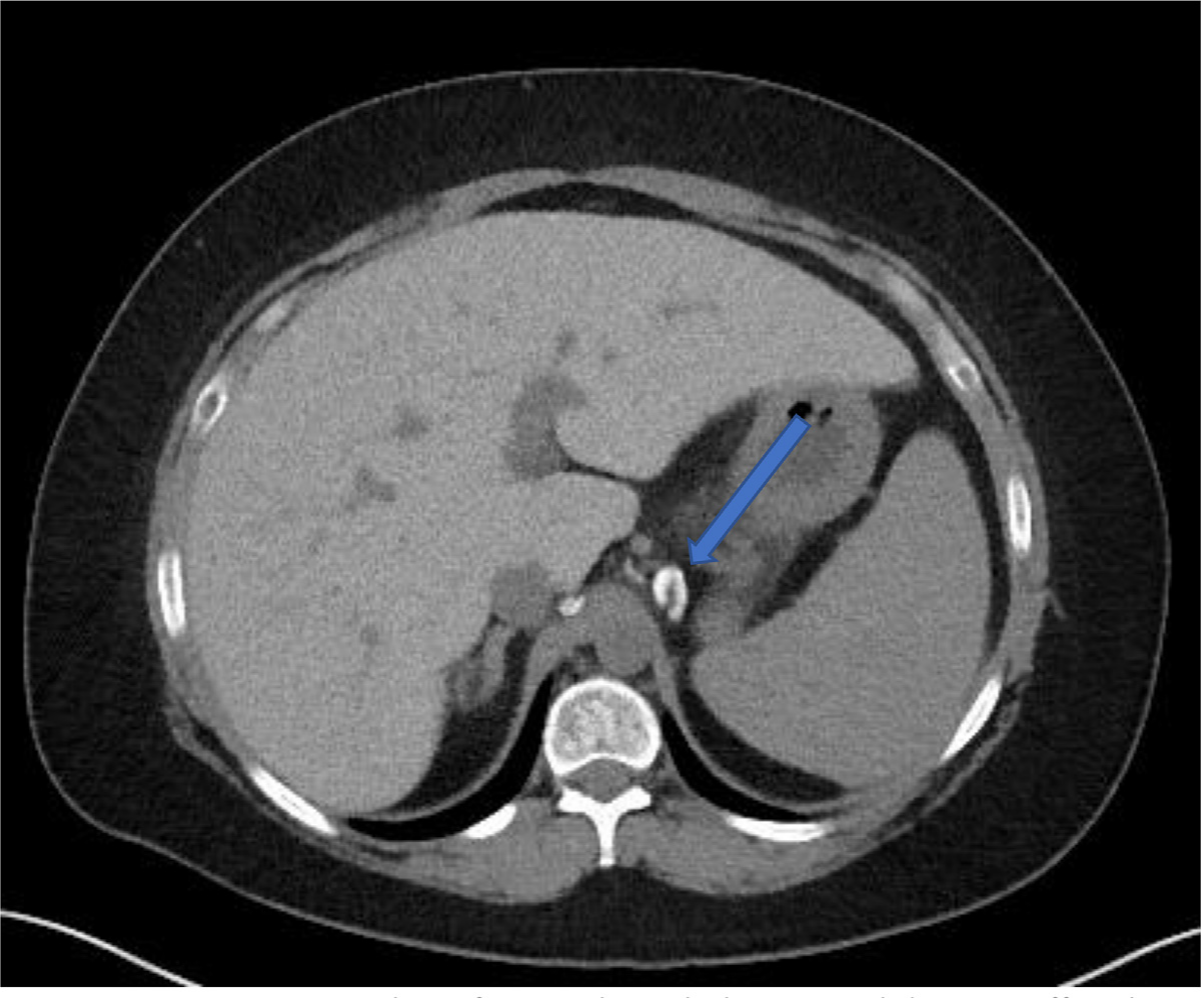
Noncontrast axial CT of image through the upper abdomen. Diffuse hyperattenuation of the liver (HU 88) compared to the spleen (HU 52). Additionally, there are high density upper abdominal lymph nodes (blue arrow). In secondary hemochromatosis, iron deposits in the reticuloendothelial system including the liver, spleen, bone marrow, and lymph nodes resulting in higher attenuation. Interesting in this case, the disease process spared the spleen which is on the upper limits of normal (40-60 HU).

**Fig. 4:**
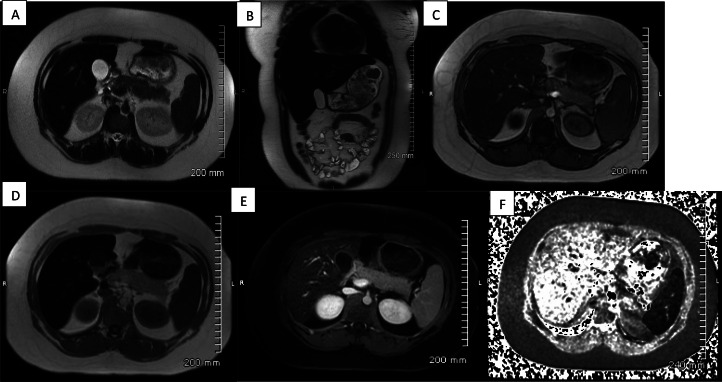
Images from a contrast-enhanced abdominal MRI from 8 years prior. T2 axial (A) and coronal (B) images show diffuse low signal throughout the liver (lower than muscle) consistent with hemochromatosis. T1 opposed-phase (C) and T1 in-phase (D) shows significant loss of signal throughout the liver with the longer echo time (in-phase images). Contrast-enhanced axial T1 image (E) again shows abnormal signal loss throughout the hepatic parenchyma relative to other abdominal organs. Axial R2* map from an iron quantification sequence (F) confirmed severe iron overload.

An MRI with and without contrast focusing on the suprasellar and pituitary region was ordered to evaluate CNS etiologies for her secondary amenorrhea. Imaging demonstrated diffuse absence of T1 and T2 signal of the pituitary gland without normal enhancement (Fig. 1, Fig. 2). The remainder of the brain MRI study was unremarkable. Review of a recent prior noncontrast CT through the abdomen demonstrated a diffusely hyperattenuating liver relative to the spleen, with Hounsfield units (HU) of 88, compared to HU of 52 respectively, as well as calcified abdominal lymph nodes (Fig. 3). Additionally, an abdominal MRI performed 8 years prior demonstrated a similar abdominal pattern of hemochromatosis with marked diffuse signal loss throughout the liver, most evident on sequences with longer echo times (T2-weighted images, and T1 in-phase with echo time of 4.4 msec relative to T1 opposed-phase with echo time of 2.1 msec) (Fig. 4). Iron quantification was also performed, showing hyperintensity on R2* relaxometry maps. The R2* mapping uses a gradient echo technique in a single breath hold to acquire multiple images at increasing echo times within the same repetition time (TR) [2]. R2* is the reciprocal of the traditional T2*, therefore the greater the iron concentration, the greater the R2* value. The use of relaxometry allows for quantification of iron concentration which in this case was a 15-19 mg/g dry weight of tissue (normal < 1.8 mg/g), corresponding to severe iron overload.

Given these findings and clinical symptoms we determined that the absence of MRI signal and enhancement within the pituitary gland was attributed to secondary hemochromatosis from a lifetime of transfusions causing pituitary dysfunction.

## Discussion

Diamond-Blackfan anemia is a rare genetic disease caused by a mutation of ribosomal synthesis resulting in p53 tumor suppressor activation that specifically affects erythropoiesis. The tumor suppressor activity results in erythroid aplasia characterized by progressive macrocytic normochromic anemia with reticulocytopenia [3,4]. It is diagnosed in childhood with approximately 90% of cases presenting in the first year of life and has an incidence of 5 cases per 1 million live births [5]. The standard treatment for Diamond-Blackfan anemia for patients, apart from stem cell transplant, is lifelong steroid and transfusion therapies. Goal transfusion therapy is hemoglobin levels of 8-10 g/dL, which may require transfusions every 4-6 weeks [6].

Similarly, our patient required consistent transfusions over her entire life and consequently developed secondary hemochromatosis. Normal serum iron and ferritin levels at our institution are 37-145 ng/mL and 13-150 ng/mL, respectively. Our patient's iron and ferritin level greatly exceeded these normal ranges at 272 mcg/dL and 15,170 ng/mL. This secondary hemochromatosis resulted in iron deposition within the pituitary gland, causing pituitary dysfunction and clinically manifesting as secondary amenorrhea.

Normally the anterior pituitary gland appears isointense on T1 and T2 with homogenous enhancement on postcontrast images. The posterior pituitary is similar except for hyperintensity on T1 due to the intrinsic T1 shortening of vasopressin [7]. In our patient there was complete absence of signal on both T1 and T2 weighted sequences and an absence of enhancement, presumably from iron deposition, which is a unique finding in the setting of secondary hemochromatosis. This lack of signal within the pituitary parenchyma is due to disruption of the magnetic field from paramagnetic properties of iron (susceptibility, also known as T2* effect).

Further corroborating this finding were the abdominal CT and MRI findings which demonstrated high attenuation of liver parenchyma on noncontrast CT due to iron deposition, signal loss on longer echo time T1- and T2-weighted images, and increased iron concentration as measured on R2* quantitative maps. Interestingly on both the CT and MRI, the patient did not have sequela of secondary hemochromatosis within the spleen or reticuloendothelial system (RES). Traditionally in secondary hemochromatosis the RES, and more specifically the spleen, follows the same imaging pattern as the liver with sparing of the pancreas. In our case the imaging findings of the RES were largely unremarkable, apart from calcified abdominal lymph nodes.

The patient was subsequently seen by her endocrinologist and placed on iron chelation therapy and oral contraception. While treatment for hemochromatosis with either iron chelation or therapeutic phlebotomy can prevent further damage and sometimes reverse early-stage effects on the pituitary, if significant damage has occurred, the pituitary function might not fully return requiring hormonal replacement therapy. Given that this patient's anemia was congenital and not a candidate for phlebotomy, the return of normal pituitary function is unlikely.

## Conclusion

This is a unique case in a patient with a history of transfusion dependent Diamond-Blackfan anemia resulting in secondary hemochromatosis and hypopituitarism, ultimately manifesting clinically as amenorrhea. Imaging characteristics of hemochromatosis are often described within the liver, RES, pancreas, and heart. Our case describes the appearance of this disease within the pituitary gland and should be considered in cases with complete absence of MRI signal within the pituitary.

## Patient consent

Written informed consent for the publication of this case report was obtained from the patient.
